# The Impact of HIV Infection on Neoadjuvant and Adjuvant Chemotherapy Relative Dose Intensity in South African Patients with Breast Cancer

**DOI:** 10.1093/oncolo/oyad056

**Published:** 2023-03-21

**Authors:** Daniel S O’Neil, Oluwatosin A Ayeni, Hayley A Farrow, Wenlong Carl Chen, Georgia Demetriou, Ines Buccimazza, Sharon Čačala, Laura W Stopforth, Maureen Joffe, Michael H Antoni, Gilberto Lopes, Yoanna S Pumpalova, Witness Mapanga, Judith S Jacobson, Katherine D Crew, Alfred I Neugut, Paul Ruff, Herbert Cubasch

**Affiliations:** Section of Medical Oncology, Department of Internal Medicine, Yale School of Medicine, Yale University, New Haven, CT, USA; Yale Cancer Center, Yale University, New Haven, CT, USA; Strengthening Oncology Services Research Unit, Department of Internal Medicine, Faculty of Health Sciences, University of the Witwatersrand, Johannesburg, South Africa; Division of Medical Oncology, Department of Internal Medicine, Faculty of Health Sciences, University of the Witwatersrand, Johannesburg, South Africa; Departments of Surgery and Radiation Oncology, Grey’s Hospital, University of KwaZulu-Natal, Pietermaritzburg, South Africa; Strengthening Oncology Services Research Unit, Department of Internal Medicine, Faculty of Health Sciences, University of the Witwatersrand, Johannesburg, South Africa; National Cancer Registry, National Health Laboratory Service, Johannesburg, South Africa; Sydney Brenner Institute for Molecular Bioscience, Faculty of Health Sciences, University of the Witwatersrand, Johannesburg, South Africa; Division of Medical Oncology, Department of Internal Medicine, Faculty of Health Sciences, University of the Witwatersrand, Johannesburg, South Africa; Department of Specialized Surgery, Inkosi Albert Luthuli Central Hospital, Durban and Ngwelezane Hospital, University of KwaZulu-Natal, Empangeni, South Africa; Departments of Surgery and Radiation Oncology, Grey’s Hospital, University of KwaZulu-Natal, Pietermaritzburg, South Africa; Department of Surgery, Ngwelezana Hospital, University of KwaZulu-Natal, Empangeni, South Africa; Departments of Surgery and Radiation Oncology, Grey’s Hospital, University of KwaZulu-Natal, Pietermaritzburg, South Africa; Strengthening Oncology Services Research Unit, Department of Internal Medicine, Faculty of Health Sciences, University of the Witwatersrand, Johannesburg, South Africa; SAMRC/Wits Developmental Pathways to Health Research Unit, Department of Paediatrics, Faculty of the Health Sciences, University of the Witwatersrand, Johannesburg, South Africa; South Africa Medical Research Council Common Epithelial Cancers Research Centre, Faculty of Health Sciences, University of Witwatersrand, Johannesburg, South Africa; Department of Psychology and Psychiatry and Behavioral Sciences, Miller School of Medicine, University of Miami, Miami, FL, USA; Sylvester Comprehensive Cancer Center, University of Miami Health System, Miami, FL, USA; Sylvester Comprehensive Cancer Center, University of Miami Health System, Miami, FL, USA; Department of Medicine, Miller School of Medicine, University of Miami, Miami, FL, USA; Herbert Irving Comprehensive Cancer Center, Columbia University Irving Medical Center, New York, NY, USA; Department of Medicine, Vagelos College of Physicians and Surgeons, Columbia University, New York, NY, USA; Strengthening Oncology Services Research Unit, Department of Internal Medicine, Faculty of Health Sciences, University of the Witwatersrand, Johannesburg, South Africa; Division of Medical Oncology, Department of Internal Medicine, Faculty of Health Sciences, University of the Witwatersrand, Johannesburg, South Africa; Herbert Irving Comprehensive Cancer Center, Columbia University Irving Medical Center, New York, NY, USA; Department of Epidemiology, Mailman School of Public Health, Columbia University, New York, NY, USA; Herbert Irving Comprehensive Cancer Center, Columbia University Irving Medical Center, New York, NY, USA; Department of Medicine, Vagelos College of Physicians and Surgeons, Columbia University, New York, NY, USA; Herbert Irving Comprehensive Cancer Center, Columbia University Irving Medical Center, New York, NY, USA; Department of Medicine, Vagelos College of Physicians and Surgeons, Columbia University, New York, NY, USA; Department of Epidemiology, Mailman School of Public Health, Columbia University, New York, NY, USA; Strengthening Oncology Services Research Unit, Department of Internal Medicine, Faculty of Health Sciences, University of the Witwatersrand, Johannesburg, South Africa; Division of Medical Oncology, Department of Internal Medicine, Faculty of Health Sciences, University of the Witwatersrand, Johannesburg, South Africa; Department of Surgery, Faculty of Health Sciences, University of the Witwatersrand, Johannesburg, South Africa

**Keywords:** breast neoplasms, HIV infections, South Africa, chemotherapy

## Abstract

**Introduction:**

In the South African Breast Cancer and HIV Outcomes (SABCHO) study, we previously found that breast cancer patients living with HIV and treated with neoadjuvant chemotherapy achieve lower rates of complete pathologic response than patients without HIV. We now assess the impact of comorbid HIV on receipt of timely and complete neoadjuvant and adjuvant chemotherapy.

**Materials and Methods:**

Since June 2015, the SABCHO study has collected data on women diagnosed with breast cancer at 6 South African hospitals. We selected a sample of participants with stages I-III cancer who received ≥2 doses of neoadjuvant or adjuvant chemotherapy. Data on chemotherapies prescribed and received, filgrastim receipt, and laboratory values measured during treatment were captured from patients’ medical records. We calculated the mean relative dose intensity (RDI) for all prescribed chemotherapies. We tested for association between full regimen RDI and HIV status, using linear regression to control for demographic and clinical covariates, and for association of HIV with laboratory abnormalities.

**Results:**

The 166 participants living with HIV and 159 without HIV did not differ in median chemotherapy RDI: 0.89 (interquartile range (IQR) 0.77-0.95) among those living with HIV and 0.87 (IQR 0.77-0.94) among women without HIV. Patients living with HIV experienced more grade 3+ anemia and leukopenia than those without HIV (anemia: 10.8% vs. 1.9%, *P* = .001; leukopenia: 8.4% vs. 1.9%, *P* = .008) and were more likely to receive filgrastim (24.7% vs. 10.7%, *P* = .001).

**Conclusions:**

HIV status did not impact neoadjuvant or adjuvant chemotherapy RDI, although patients with breast cancer living with HIV experienced more myelotoxicity during treatment.

## Teaser

Results of the South African Breast Cancer and HIV Outcomes (SABCHO) study showed that patients with breast cancer living with HIV and treated with neoadjuvant chemotherapy achieve lower rates of complete pathologic response than patients without HIV. This article assesses the impact of comorbid HIV on receipt of timely and complete neoadjuvant and adjuvant chemotherapy.

**Implications for Practice**

Among patients with breast cancer receiving neoadjuvant chemotherapy, women living with HIV (WLWH) achieve fewer pathologic complete responses than women without HIV. It is unknown whether WLWH experience more frequent chemotherapy dose reductions and delays, which could explain the differences in response rates. This study compared chemotherapy relative dose intensity (RDI) in South African patients with breast cancer with and without HIV. RDI did not differ, but WLWH demonstrated more myelotoxicity and required more filgrastim. The findings suggest that it is feasible to deliver appropriate dose intensity chemotherapy to WLWH and that alternative explanations are needed for these women’s poorer chemotherapy responses.

## Introduction

As life expectancy for people living with HIV increases worldwide, more women living with HIV (WLWH) are being diagnosed with age-related diseases, including breast cancer.^1-3^ Although WLWH do not seem to be at higher risk than others of developing breast cancer, those who do suffer approximately 50% higher mortality than patients with breast cancer without HIV.^4-8^

The causes of these survival disparities are not fully understood. WLWH appear to be diagnosed with more advanced cancers and experience delays in starting cancer therapy in the USA.^8,9^ However, three-quarters of patients with breast cancer with comorbid HIV live in sub-Saharan Africa (SSA), where they experience similar survival disparities without any apparent differences in breast cancer treatment quality.^10-15^ Little is known about the extent to which HIV infection might directly affect breast cancer prognosis by impacting anti-tumor immunity or promoting a chronic inflammatory response.

We have previously shown that, in a cohort of 715 South African patients with breast cancer drawn from the South African Breast Cancer and HIV Outcomes (SABCHO) study, the rate of pathologic complete response (pCR) following neoadjuvant chemotherapy was 52% lower among WLWH than among women without HIV.^16^ At the time, we could not say whether the WLWH were less tolerant of chemotherapy and more prone to experience chemotherapy dose reductions and delays, a difference that might have explained the differences in response rates.

Chemotherapy relative dose intensity (RDI) is a single measure that captures the actual total dose and duration of chemotherapy delivered, expressed as a proportion of the ideal prescribed dose and duration.^17^ Thus, dose reductions and delays result in lower RDI. Analyses of randomized-controlled trials and observational studies of breast cancer patients have shown that neoadjuvant and adjuvant chemotherapy RDI < 0.85 is associated with decreased progression-free and overall survival.^17-20^ Earlier studies were conducted almost exclusively in high-income countries and included few WLWH. However, in a recent study of Botswanan patients with breast cancer, 24% of whom were also living with HIV, neoadjuvant chemotherapy RDI <0.85 also showed association with lower pCR rates.^21^

We therefore compared South African patients with breast cancer with and without HIV with respect to neoadjuvant and adjuvant chemotherapy dose delays and reductions, as captured by RDI. Secondarily, we compared the types of chemotherapy regimens prescribed to these women and documented toxicities during treatment.

## Methods

### Context

South Africa is an upper-middle-income country with drastic income inequality; 56% of the population lives beneath the national poverty line, which was 890 ZAR (~58 USD) per month in 2021.^22,23^ Approximately 85% of the population is partially or entirely dependent on public healthcare services.^24^ HIV care is universally available, and HIV prevalence is 21% among Black African women.^25^ In most provinces, public ­tertiary-level hospitals offer diagnostic and therapeutic breast cancer care (eg, pathology, advanced imaging, surgery, radiation, chemotherapy, and endocrine therapy), but they vary in the timeliness and quality of care they provide.^10^

Since 2015, the SABCHO cohort study has been prospectively enrolling recently diagnosed female patients with breast cancer over 18 years old from 6 public hospitals in provinces.^26^ We have collected detailed demographic, clinical, and ongoing outcomes data from more than 3500 women to date, and have confirmed an overall survival disparity between patients with breast cancer with and without comorbid HIV infection.^14^ Baseline SABCHO data include receipt of neoadjuvant or adjuvant chemotherapy but not amounts or dates for individual doses.

### Participants

For this analysis, we retroactively enrolled a sub-group of participants from the wider SABCHO cohort who were all of Black African race; had a histologically confirmed new diagnosis of breast cancer between June 1, 2015 and June 30, 2019; presented with AJCC 7th edition stages I-III disease; and received at least 2 doses of curative-intent chemotherapy in either the neoadjuvant or the adjuvant setting. We also planned to exclude participants who received “sandwich” chemotherapy, defined as a single planned regimen interrupted by breast surgery midway through treatment, as the pause in treatment for surgery confounds the calculation of RDI. Of note, we did not encounter any potential participants who received these sorts of “sandwich” regimens. We included patients who received chemotherapy at Charlotte Maxeke Johannesburg Academic Hospital (CMJAH), in the province of Gauteng, and Grey’s Hospital, in the province of KwaZulu-Natal, because those 2 hospitals had complete data available at the time of analysis. On a *post hoc* basis, we excluded participants who received carboplatin or methotrexate because so few participants received those drugs that comparisons by HIV status were not possible.

### Procedures and Data Collection

A list of all potentially eligible SABCHO participants from CMJAH and Grey’s Hospital was prepared using data from the SABCHO study database. Those lists were then divided by HIV status and placed in a random order. Study staff sequentially reviewed patients on both lists, accessing their medical records to confirm eligibility for this study. If a participant was eligible, the following data were captured from their medical record: the first prescribed neoadjuvant or adjuvant chemotherapy regimen; height, and weight at the start of chemotherapy; the type, dose, and date of all neoadjuvant and adjuvant chemotherapy delivered; laboratory data collected immediately before and up to 21 days after chemotherapy administration, including complete blood counts, metabolic panels, and hepatic function tests; and receipt of filgrastim with each chemotherapy dose. We stored these data in a REDCap database hosted by the University of Witwatersrand.^27^ SABCHO participants living with and without HIV were enrolled in approximately equal rates, and enrollment into this study continued on an ongoing basis until the total enrolled patients surpassed the sufficient sample size described below.

For each enrolled patient, we also extracted previously collected SABCHO study data on age at breast cancer diagnosis, marital and educational status, household wealth, breast cancer stage and grade, estrogen and progesterone receptor (ER/PR) status, human epidermal growth factor receptor 2 status (HER2), performance status, and vital status.

### Outcomes and RDI Calculations

Our primary outcome was overall neoadjuvant or adjuvant chemotherapy relative dose intensity (RDI). RDI describes the proportion of the standard dose intensity delivered and was calculated using the following formula:


$$\begin{array}{l} \frac{\mathrm{Sum}\;\mathrm{of}\;\mathrm{All}\;\mathrm{Delivered}\;\mathrm{Doses}/\mathrm{Total}\;\mathrm{Days}\;\mathrm{to}\;\mathrm{Deliver}\;\mathrm{All}\;\mathrm{Doses}\;\mathrm{Given}}{\mathrm{Standard}\;\mathrm{Single}\;\mathrm{Dose}/\mathrm{Standard}\;\mathrm{Days}\;\mathrm{Between}\;\mathrm{Doses}} \end{array}$$

The RDI of each chemotherapeutic agent was calculated separately, and we used the unweighted mean RDI of all agents in the *first* planned treatment regimen as the overall RDI.^28^ If a regimen was changed midway through treatment, we did not include substitute agents in the final RDI calculation.

Standard dosing amounts, cycle lengths, and cycle numbers were based on existing institutional protocols (Table 1). We consulted treatment notes to determine the initial chemotherapy plan when various cycle numbers could be considered standard (eg, 4 or 6 cycles of combination docetaxel and cyclophosphamide).^29-31^ When any chemotherapy doses were missed entirely, a dose of zero and a standard cycle length were assigned to each missed dose. We did not want instances of excessive chemotherapy dosing to counterbalance instances of underdosing, so when delivered dose amounts for an agent exceeded standard protocol doses, we used the full standard dose as the delivered dose. For the same reason, chemotherapy doses given beyond the number pre-specified by the planned regimen were excluded from RDI calculations.

**Table 1.** Standard neoadjuvant and adjuvant chemotherapy regimens for breast cancer at Charlotte Maxeke Johannesburg Academic Hospital (Gauteng) and Grey’s Hospital (KwaZulu-Natal).

Regimen	Drugs	Dose (mg/m^2^)	Cycle length (days)	Cycle number
AC	Doxorubicin/Epirubicin	60/75	21	4
	Cyclophosphamide	600	21	4
AC-T	Doxorubicin/Epirubicin	60/75	21	4
	Cyclophosphamide	600	21	4
	Paclitaxel/Docetaxel	175/75	21	4
FAC	Fluorouracil	500	21	6
	Doxorubicin/Epirubicin	50/75	21	6
	Cyclophosphamide	500	21	6
FAC-T	Fluorouracil	500	21	4
	Doxorubicin/Epirubicin	50/75	21	4
	Cyclophosphamide	500	21	4
	Paclitaxel/Docetaxel	175/75 or 100^1^	21	4
TC	Cyclophosphamide	500	21	4 or 6
	Paclitaxel/Docetaxel	175/75	21	4 or 6
TAC	Doxorubicin/Epirubicin	50/75	21	6
	Cyclophosphamide	500	21	6
	Paclitaxel/Docetaxel	175/75	21	6
T	Paclitaxel/Docetaxel	175/75	21	6

^1^Varied by treating hospital.

Abbreviations: A, anthracycline; C, cyclophosphamide; F, fluorouracil; T, Taxane.

Drug stockouts were common and sometimes necessitated drug substitutions, such as docetaxel instead of paclitaxel or epirubicin instead of doxorubicin. We did not want a stockout to be reported as reduced RDI if a drug was replaced with a drug of the same class. For that reason, doxorubicin and epirubicin were both treated as a single agent, “anthracycline,” with dose amounts transformed to a standard value for each standard regimen. Similarly, paclitaxel and docetaxel were both treated as “taxane.”

### Sample Sizes and Analysis

Estimating RDI’s standard deviation at 0.15 and using an alpha of 0.05, enrolling 2 groups of 143 participants would give 80% power to detect a 0.05 difference in RDI via a 2-sample *t*-test.

We described demographics, breast cancer clinical data, chemotherapies received, and chemotherapy-concurrent toxicity using counts and percentages and used chi-square testing to compare these characteristics by HIV status.

We computed the medians and interquartile ranges (IQRs) of the RDIs of individual chemotherapy agents and the overall regimen and compared these values by HIV status using univariate linear regression. For full regimen RDI, univariate linear regression was also performed for all measured demographic and breast cancer clinical characteristics. Any characteristic with a possible impact on RDI in this analysis, defined as a *P*-value ≤ 0.1, was included in a multivariable linear regression model along with HIV status. Rates of Common Terminology Criteria for Adverse Events v5.0 (CTCAE) grade 3 or higher anemia, leukopenia, neutropenia, thrombocytopenia, alkaline phosphatase elevation, and creatinine elevation were calculated from the raw laboratory data and compared using chi-square tests.

## Results

Our initial list of potentially eligible SABCHO participants included 1479 women who received chemotherapy at CMJAH (1123 women without HIV and 356 women living with HIV) and 303 women who received chemotherapy at Grey’s Hospital (207 women without HIV and 96 women living with HIV). Chemotherapy data were collected on the first 330 participants who were deemed eligible on chart review. During analysis, 5 of these women were subsequently determined to be ineligible and were excluded. One was excluded because she had been diagnosed with in situ carcinoma only; 3 were excluded for having received carboplatin or methotrexate; and 1 was excluded because she received zero doses of her originally prescribed treatment. Of the remaining 325 subjects, 166 (51%) were WLWH, and 159 (49%) were uninfected.

WLWH were younger, less likely to be married, and more likely to have some formal education than those without HIV (Table 2). The 2 groups did not differ in household wealth, treating hospital, breast cancer stage, grade, ER and PR status, HER2 status, or ECOG performance status before or after chemotherapy.

**Table 2.** Demographic and breast cancer clinical characteristics.

Characteristic	HIV-negative (*N* = 159)		HIV-positive (*N* = 166)		*P*-value^1^
	*n*	%	*n*	%	
Age (years)					<.0001
<40	23	14.5	36	21.7	
40-50	43	27.0	89	53.6	
50-60	45	28.3	31	18.7	
60-70	30	18.9	9	5.4	
≥70	18	11.3	1	0.6	
Marital status					.02
Married	58	36.5	40	24.2	
Unmarried	101	63.5	125	75.8	
Education					.04
None/some primary	32	20.1	15	9.1	
Primary	60	37.7	69	42.1	
Secondary	51	32.1	65	39.6	
Post-secondary	16	10.1	15	9.1	
Wealth index (percentile)					.11
<20th	49	30.8	49	29.5	
20-40th	26	16.4	46	27.7	
40-60th	29	18.2	21	12.7	
60-80th	26	16.4	20	12.0	
≥80th	29	18.2	30	18.1	
Treating hospital					.75
CMJAH	94	59.1	101	60.8	
GH	65	40.9	65	39.2	
Year of diagnosis					.02
2015	22	13.8	19	11.4	
2016	34	21.4	59	35.5	
2017	33	20.8	41	24.7	
2018	50	31.4	32	19.3	
2019	20	12.6	15	9.0	
Stage					.24
I	4	2.5	10	6.1	
II	54	34.0	49	29.7	
III	101	63.5	106	64.2	
Grade					.53
1	12	7.7	14	8.6	
2	74	47.7	86	53.1	
3	69	44.5	62	38.3	
ER/PR status					.46
Positive	126	79.7	126	76.4	
Negative	32	20.3	39	23.6	
HER2 status					.20
Positive	35	22.2	41	24.8	
Negative	108	68.4	99	60.0	
Equivocal	15	9.5	25	15.2	
Pre-chemotherapy ECOG performance status					.92
0	118	74.2	121	72.9	
1	39	24.5	43	25.9	
2	2	1.3	2	1.2	
Post-chemotherapy ECOG performance status					.74
0	119	75.3	121	75.2	
1	35	22.2	38	23.6	
2	4	2.5	2	1.2	
Missing	1		5		
Antiretroviral medication use at enrollment					—
Yes	—	—	139	83.7	
No	—	—	27	16.3	
CD4 cell count at enrollment (cells/mL)					—
<500	—	—	75	46.3	
≥500	—	—	87	53.7	
HIV viral load (viral copies/mL)					—
≤50	—	—	61	53.0	
>50	—	—	54	46.0	

^1^Chi-square testing.

Abbreviations: CMJAH, Charlotte Maxeke Johannesburg Academic Hospital; ER/PR Estrogen receptor/progesterone receptor; GH, Grey’s Hospital; HER2, human epidermal growth factor receptor 2.

**Figure 1.**
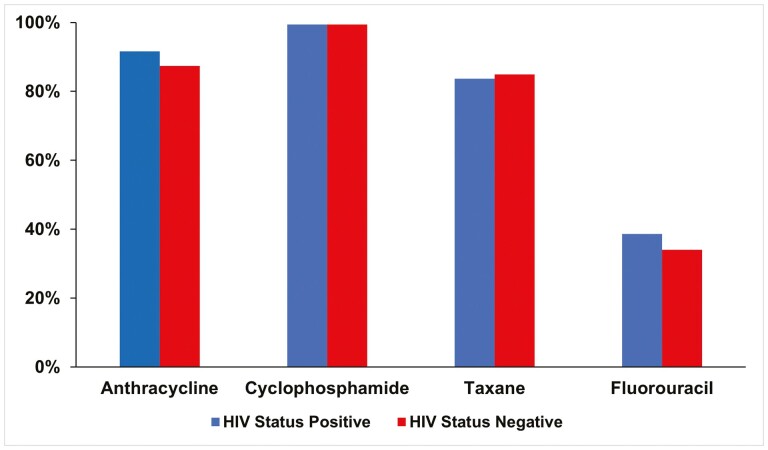
Neoadjuvant and adjuvant chemotherapy agents prescribed to patients with breast cancer at Charlotte Maxeke Johannesburg Academic Hospital (Gauteng) and Grey’s Hospital (KwaZulu-Natal), by HIV status.

The most commonly prescribed neoadjuvant or adjuvant chemotherapy regimen was a combination of anthracycline and cyclophosphamide followed by a taxane (47.8% of women without HIV and 45.8% of WLWH), and the next most common was a combination of fluorouracil, anthracycline, and cyclophosphamide followed by a taxane (23.9% of women without HIV and 28.3% of WLWH) (Table 3). The 2 groups did not differ in prescribed chemotherapy types (Fig. 1).

**Table 3.** Neoadjuvant and adjuvant chemotherapy regimens prescribed.

Plan	HIV negative (*N* = 159)		HIV positive (*N* = 166)	
	*n*	%	*n*	%
AC	8	5.0	10	6.0
AC-T	76	47.8	76	45.8
FAC	16	10.1	17	10.2
FAC-T	38	23.9	47	28.3
T	1	0.6	1	0.6
TAC	1	0.6	2	1.2
TC	19	11.9	13	7.8

Abbreviations: A, anthracycline; C, cyclophosphamide; F, fluorouracil; T, Taxane.

The median RDIs of individual chemotherapy types ranged from 0.89 to 0.92 in women without HIV and from 0.87 to 0.92 in WLWH. The groups did not differ in anthracycline, cyclophosphamide, taxane, or fluorouracil RDI by HIV status (Table 4). The full regimen RDI for the entire cohort was 0.88 (IQR 0.77-0.94). Among women without HIV, median RDI was 0.87 (IQR 0.77-0.94), and among WLWH, it was 0.89 (IQR 0.77-0.95) (*P* = 0.70). Overall, 137 (42.2%) women received chemotherapy with RDI < 0.85%, including 70 WLWH and 67 without HIV.

**Table 4.** Relative dose intensity of neoadjuvant and adjuvant chemotherapy agents.

Agent	HIV negative (*N* = 159)		HIV positive (*N* = 166)		*P*-value^1^
	Median	IQR	Median	IQR	
Anthracycline	0.90	0.78-0.97	0.88	0.77-0.98	0.71
Cyclophosphamide	0.91	0.79-0.97	0.88	0.79-0.97	0.70
Taxane	0.92	0.75-0.98	0.92	0.78-0.98	0.92
5-FU	0.89	0.81-0.98	0.87	0.75-0.96	0.36

^1^Wilcoxon rank sum test.

Abbreviation: IQR, interquartile range.

The only factors associated with overall RDI on univariate analysis were ER/PR status (ER/PR positive: 0.90, IQR 0.79-0.95 and ER/PR negative: 0.84, IQR 0.73-0.93, *P* = .02) and HER2 status (HER2 positive: 0.90, IQR 0.80-0.97, HER2 equivocal: 0.89, IQR 0.80-0.95, and HER2 negative: 0.87, IQR 0.76-0.94, *P* = .02) (Table 5). ECOG performance status prior to chemotherapy was marginally associated with RDI. In a model that included ER/PR status, HER2 status, and ECOG performance status, HIV status was not associated with overall RDI.

**Table 5.** Relative dose intensity in demographic and clinical subgroups.

	Median	IQR	Unadjusted *P*-value^1^	Adjusted *P*-value^2^
All women (*N* = 325)	0.88	0.77-0.94	—	—
Age (years)			0.88	—
<40	0.87	0.77-0.95		
40-50	0.87	0.76-0.95		
50-60	0.89	0.81-0.94		
60-70	0.87	0.76-0.93		
≥70	0.92	0.76-0.96		
Marital status			0.98	—
Married	0.89	0.78-0.94		
Unmarried	0.88	0.77-0.95		
Education			0.94	—
None or primary only	0.87	0.77-0.95		
Secondary or beyond	0.87	0.77-0.95		
Wealth index (percentile)			0.89	—
<20th	0.89	0.78-0.95		
20-40th	0.86	0.75-0.94		
40-60th	0.88	0.77-0.93		
60-80th	0.91	0.76-0.96		
≥80th	0.86	0.79-0.95		
Treating hospital			0.24	—
CMJAH	0.88	0.76-0.95		
GH	0.88	0.79-0.94		
Year of diagnosis			0.12	—
2015-2016	0.9	0.80-0.95		
2017-2019	0.87	0.77-0.94		
Stage			0.14	—
I-II	0.87	0.74-0.94		
III	0.87	0.78-0.95		
Grade			0.29	—
1-2	0.88	0.76-0.94		
3	0.89	0.80-0.95		
ER/PR status			0.02	0.04
Positive	0.9	0.79-0.95		
Negative	0.84	0.73-0.93		
HER2 status			0.02	0.047
Positive	0.9	0.80-0.97		
Equivocal	0.89	0.80-0.95		
Negative	0.87	0.76-0.94		
Pre-chemotherapy ECOG performance status			0.07	0.08
0	0.87	0.76-0.94		
1	0.90	0.81-0.95		
2	0.93	0.80-0.97		
HIV status			0.7	0.70
Positive	0.89	0.77-0.95		
Negative	0.87	0.77-0.94		

^1^Univariable linear regression.

^2^Multivariable linear regression with ER/PR status, HER2 status, performance status, and HIV status included as covariates.

Abbreviations: CMJAH, Charlotte Maxeke Johannesburg Academic Hospital; ER/PR, estrogen receptor/progesterone receptor; GH, Grey’s Hospital; HER2, human epidermal growth factor receptor 2; IQR, interquartile range.

However, WLWH had more documented myelotoxicity than uninfected women during chemotherapy receipt. Among participants for whom data was available, 3 (1.9%) women without HIV and 18 (10.8%) WLWH had CTCAE grade ≥3 anemia (*P* = .001), and 3 (1.9% women without HIV and 14 (8.4%) WLWH had grade ≥3 leukopenia (*P* = .008). In addition, 17 (10.7%) women without HIV and 41 (24.7%) WLWH received at least one dose of filgrastim with their chemotherapy (*P* = .001). (Table 6). The 2 groups did not differ in grade ≥3 neutropenia, thrombocytopenia, alkaline phosphatase elevation, or creatinine elevation.

**Table 6.** Measured toxicity (grade ≥3) occurring during neoadjuvant or adjuvant chemotherapy.

Toxicity	HIV negative		HIV positive		*P*-value^1^
	*n*	%^2^	*n*	%^2^	
Anemia (*N* = 325)	3	1.9	18	10.8	.001
Leukopenia (*N* = 325)	3	1.9	14	8.4	.008
Neutropenia (*N* = 127)	9	13.9	9	14.5	.91
Thrombocytopenia (*N* = 325)	1	0.6	1	0.6	.98
Alkaline phosphatase elevation (*N* = 183)	0	0.0	1	1.0	.35
Creatinine elevation (*N* = 262)	0	0.0	1	0.8	.31

^1^Chi-square test.

^2^Not all patients had laboratory data for all toxicity types. Percentages are based on the numbers of patients who had laboratory data for each toxicity. Availability of laboratory data did not differ based on HIV status.

In exploratory univariate analyses of only participants living with HIV, no significant association was found between overall RDI and either HIV viral load ≤50 copies/mL, CD4 count ≥500 cells/mL, or antiretroviral medication (ARV) use at SABCHO study enrollment. A weak trend toward lower overall RDI was noted in participants not using ARVs (non-users: 0.80, IQR 0.72-0.95 and ARV users: 0.90, IQR 0.79-0.95, *P* = .15).

## Discussion

In our subgroup analysis of SABCHO participants, we found no differences in prescribed treatments or RDI for neoadjuvant and adjuvant chemotherapy between South African patients with breast cancer with and without comorbid HIV infection. Further, RDI was not associated with age, educational status, or household wealth. Median RDI was slightly higher in women whose breast cancers were ER/PR positive (0.9 vs. 0.84) and in those whose cancers were HER2 positive (0.9 vs. 0.87) than in others. Despite incomplete data, we did find evidence that WLWH were more prone than others to experience dose-limiting myelotoxicities, such as anemia and leukopenia, and to require filgrastim support during treatment.

Notably, the median RDI for the entire cohort was 0.88 and was below 0.85 in 42% of participants. In an analysis of US patients with cancer treated from 1997 to 2000 in community practices, 56% received an RDI below 0.85.^32^ However, in the mid-2000s, a repeat study of the same practices found just 16% of patients received an RDI <0.85 and studies from Louisiana and California found 28%-30% of patients receiving an RDI <0.85.^18,20,33^ At present, chemotherapy dose reductions and delays appear to be more common in South Africa than in the US, likely reflecting structural health system challenges, such as drug stock-outs, understaffed infusion clinics, reduced access to supportive medications, and socioeconomic pressures on patients that disrupt adherence to their treatment schedule. If HIV infection potentially impacts chemotherapy tolerance, we may not have detected that effect because of the low RDI overall in our patient population.

We did not find any evidence that differences in chemotherapy tolerance or receipt explain the previously documented differences in chemotherapy response between patients with breast cancer with and without HIV.^16^ However, comorbid HIV infection may directly impact the likelihood of a pCR. Increasing density of tumor-infiltrating lymphocytes is associated with increased pCR rates in both hormone receptor positive and negative breast cancer.^34-36^ Virally suppressed people living with HIV demonstrate exhaustion of T cells, reducing their effector functions.^37^ Increased ratio of exhausted type infiltrating T cells has been associated with poorer overall survival among Black American patients with breast cancer.^38^ Perhaps HIV has a similar effect on patients with breast cancer, reducing tumor sensitivity to chemotherapy and likelihood of a pCR. Studies of intra-tumoral immune-related gene expression are ongoing for SABCHO patients and will hopefully help to clarify the effect, if any, of comorbid HIV infection on the innate tumor immune response.

Our findings conflict with a recent report from Botswana, which found that 26 patients with breast cancer living with HIV experienced significantly lower neoadjuvant chemotherapy RDI than 84 patients without HIV (mean RDI: 0.70 vs. 0.81).^21^ However, that study also found comparable rates of myelotoxicity and reported that granulocyte colony-­stimulating factor was not typically used. These findings add support to the hypothesis that our population’s easier access to filgrastim allowed WLWH to overcome a tendency toward myelotoxicity and receive chemotherapy doses equivalent to women without HIV. Without the ability to treat leukopenia, practitioners may prophylactically dose reduce. Indeed, in Botswana, a higher proportion of WLWH received reduced dose chemotherapy during their first cycle (24% vs. 14%).

Only a small fraction of patients with breast cancer within the US and Europe are WLWH. Detailed chemotherapy dosing data is rarely available for populations with large numbers of patients with breast cancer living with HIV. This study provides the largest comparison of modern, curative-intent chemotherapy RDI between patients with cancer with and without HIV of which we are aware. Our study population, Black South African women, is also representative of the global population most likely to suffer from comorbid breast cancer and HIV. However, our findings may not be generalizable to WLWH in high-income countries, where baseline neoadjuvant and adjuvant chemotherapy RDI tends to be higher and structural barriers to high quality breast cancer care may be more unequally distributed between women with and without HIV.

Our study has additional limitations. We opted to exclude women who received only a single dose of planned chemotherapy because we believed that such discontinuation of treatment was more often the result of non-adherence and treatment abandonment than severe chemotherapy intolerance that could not be managed with dose reductions. However, we do not have data on whether more WLWH were excluded under this criterion and cannot completely rule out selection bias. Given that poorer chemotherapy tolerance was not associated with HIV in the women we did enroll, it seems unlikely that any bias from excluding women who received only one dose of chemotherapy would change our overall findings. We also did not have detailed data on which ARV medications patients were using at the time of chemotherapy receipt, so we were not able to evaluate for interactions between RDI and specific ARV classes. Finally, 20 of our 325 participants were prescribed combination doxorubicin and cyclophosphamide alone without a taxane or a taxane alone without other chemotherapy. All such participants had stage II or III cancer, for which those regimens may have been insufficient. Although WLWH were not more likely to receive those regimens than other participants, we should remember that receipt of full RDI chemotherapy is not necessarily synonymous with receipt of adequate adjuvant therapy.

Many South African patients with breast cancer, including those living with HIV, are receiving inadequate chemotherapy. Behavioral interventions targeting patient-based treatment delays and logistical/operational interventions targeting clinic-based delays may help. Patients would likely derive a significant survival benefit from improved neoadjuvant and adjuvant chemotherapy adherence, especially those with hormone receptor negative cancers. Further study of HIV’s impact on tumor biology and chemotherapy pharmacokinetics may explain our earlier findings of poor chemotherapy response among patients with breast cancer living with HIV.

## Data Availability

The data underlying this article will be shared on reasonable request to the corresponding author.
